# Gastrointestinal bleeding due to idiopathic early onset of vitamin K deficiency bleeding in a girl baby 50 min after birth: a rare case

**DOI:** 10.1186/s12887-022-03744-x

**Published:** 2022-11-17

**Authors:** Harapan Parlindungan Ringoringo, Katherine Richel Tambunan, Fajar Khalis Ananda, Felynawati Nawati, Yanuar Nusca Permana

**Affiliations:** 1 Department of Child Health, Faculty of Medicine, Lambung Mangkurat University – RSD Idaman Banjarbaru, Jl. Citra Megah Raya III No.14 RT 007/RW 002, Kelurahan Loktabat Utara, Kecamatan Banjarbaru Utara , South Kalimantan 70712 Banjarbaru, Indonesia; 2 Mayapada Hospital Tangerang, Banten, Indonesia; 3 RSD Idaman Banjarbaru, Banjarbaru, South Kalimantan Indonesia

**Keywords:** Gastrointestinal bleeding, Vitamin K deficiency bleeding, Early-onset, Idiopathic, Case report

## Abstract

**Background:**

The incidence of early-onset vitamin K deficiency bleeding (VKDB) in at-risk neonates who did not receive vitamin K supplementation varied from 6 to 12%. This case report aims to show that VKDB can occur abruptly after birth despite vitamin K1 1 mg IM being given immediately after birth.

**Case presentation:**

A term female baby was born through vaginal delivery of a 28 years old mother, G1P0A0, 39–40 weeks gestation with normal APGAR score, and birth weight was 3445 g, birth length was 52 cm. During pregnancy, the mother did not take any drugs except vitamins. There are no abnormalities on the baby’s physical examination. The anus is patent. Immediately after birth, the baby received a vitamin K1 1 mg intramuscularly. Abruptly, 50 min after delivery, there was meconium with lots of fresh blood. Laboratory results showed hemoglobin, 19.6 g/dL; leukocytes, 25,010/uL; platelets, 390,000/uL, with increased PT and aPTT. A peripheral blood smear showed a normal blood morphology. When 7 h old, the baby had much hematochezia. Laboratory results showed decreased hemoglobin to 17.5 g/dL and increased PT, aPTT, and INR. No abnormalities were found on the babygram and abdominal ultrasound. The working diagnosis was gastrointestinal bleeding due to idiopathic early-onset VKDB. The baby received vitamin K1 2 mg IM, Fresh Frozen Plasma, and a Packed Red Cells transfusion. The patient returned home in good clinical condition.

**Conclusion:**

Vitamin K1 1 mg IM prophylaxis should be given immediately after birth to prevent early-onset VKDB. In addition, pregnant women who receive drugs that interfere with vitamin K metabolism (anti-epileptic drugs, anti-tuberculosis drugs, vitamin K antagonist drugs) should be given prophylactic vitamin K1, 20 mg/d orally, for at least two weeks before the expected time of delivery.

## Background

VKDB is defined as a bleeding disorder caused by insufficient activity of vitamin K-dependent coagulation factors in which the coagulation is promptly corrected by vitamin K supplementation. The process of hemostasis is a complex mechanism involving local reactions of blood vessels (vascular phase), the activity of platelets (platelet phase), and the interaction of several specific coagulation factors circulating in the blood (plasma phase). If one of the three factors is disturbed, there will be a disturbance in the hemostasis process, whose clinical manifestations are bleeding. Acquired clotting disorders can be caused by a deficiency of vitamin K-dependent clotting factors, liver disease, accelerated breakdown of coagulation factors, and coagulation inhibitors [1]. Vitamin K is essential for the activity of several carboxylase enzymes in liver cells. Vitamin K is required to synthesize and activate coagulation factors II, VII, IX, X, proteins C, and S. The half-lives of vitamin K-dependent coagulation factors are short. Therefore, vitamin K deficiency may lead to neonatal VKDB  [2–5].

The incidence of early-onset vitamin K deficiency bleeding (VKDB) in at-risk neonates who did not receive vitamin K supplementation is 6–12% [6, 7]. Among cases that did not receive vitamin K administration before delivery, the occurrence of early-onset VKDB is 50% [6]. The usual bleeding sites are the head (cephalohematoma, intracranial), intra-thoracic, intra-abdominal, or gastrointestinal tract. The severity of the clinical presentation may be related to the type of maternal treatment that interferes with vitamin K activity [8]. The prognosis of early-onset VKDB is poor because of a high incidence of intracranial hemorrhage, 25% [9, 10]. Currently, VKDB is usually categorized by etiology as idiopathic and secondary. In secondary VKDB, there is an underlying cause, usually an undiagnosed disease. Such as hereditary hepatobiliary/ malabsorptive disease or the effect of drugs given to the mother. Then, by time of onset, VKDB is divided into three types, namely: early-onset VKDB (0–24 h), classic-onset (2–7 days), and late-onset (2–12 weeks) [8]. The hallmark of VKDB is that the bleeding stops immediately after administering vitamin K1. This case report shows that VKDB can occur shortly after birth, and the bleeding stops immediately after administering vitamin K1.

## Case presentation

A mother of 28 years old, G1P0A0, 39–40 weeks gestation, came to the hospital for vaginal water discharge. In the family, there is no bleeding disease history. The laboratory results showed hemoglobin 7 g/dL, leucocyte 9,780/uL, platelets 243,000/uL, hematocrit 23.9%; PT, aPTT, AST, and ALT were within normal limits. The mother’s blood group was O, Rhesus (+). Then Screening tests for syphilis, HIV, B Hepatitis, RT PCR Sars-Cov-2 were non-reactive. During pregnancy, the mother did not take any drugs except vitamins.

**Fig. 1:**
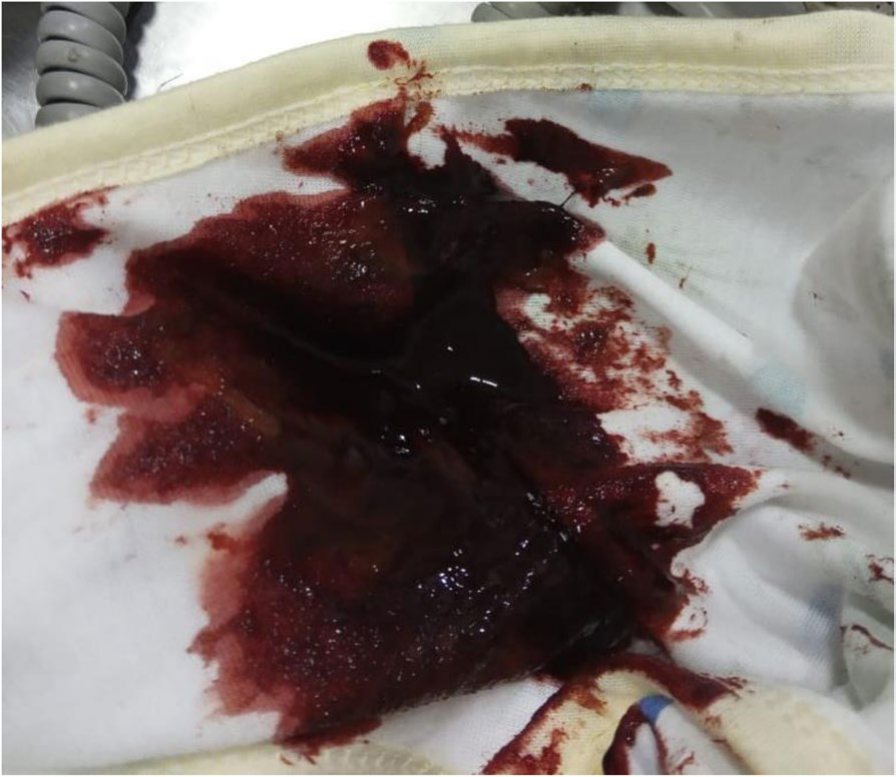
Haematochezia of the baby

**Fig. 2:**
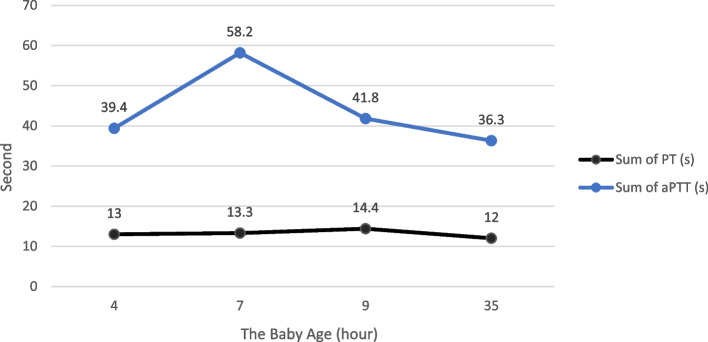
Baby’s PT and aPTT monitoring chart from the beginning until the discharge

**Fig. 3:**
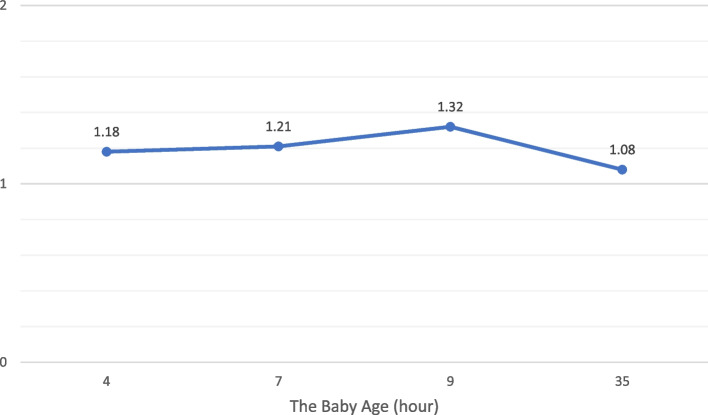
Baby’s INR monitoring chart from the beginning until the discharge

A term female baby was born through vaginal delivery with an APGAR score was eight at 1 min and nine at 5 min. The baby’s birth weight was 3445 g, length 52 cm, head circumference 33 cm with blood group O, Rhesus (+). Physical examination showed no abnormalities of the eyes, ears, nose, throat, and mouth. Heart and lungs were within normal limits, with no organomegaly or lymphadenopathy. There was no congenital anomaly. The anus is patent. Immediately after birth, the baby received a 1 mg IM injection of vitamin K1. Abruptly, 50 min after delivery, there was meconium with lots of fresh blood (Haematochezia) (see Fig. 1). Laboratory examination showed haemoglobin, 19.6 g/dL; leukocytes, 25,010/uL; platelets, 390,000/uL; and haematocrit, 57,4%. A peripheral blood smear showed a normal blood morphology. PT 13.0 s (Normal : 11–15 s), aPTT 39.4 s (Normal 25–35 s). Follow-up of PT, aPTT and INR can be seen in Figs. 2 and 3.

When the baby was 7 h old, the baby had much haematochezia. Laboratory examination showed haemoglobin, 17.5 g/dL; leukocytes, 21,180/uL; platelets, 381,000/uL; and haematocrit, 51.9%, and increasing PT, aPTT and INR. No abnormalities were found on the babygram and abdominal ultrasound. Currently, checking the levels of clotting factors II, VII, IX, and X cannot be done because the reference laboratory does not have examination facilities. The working diagnosis was gastrointestinal bleeding due to idiopathic early-onset VKDB. The baby received vitamin K1 2 mg IM, 2 × 60 ml Fresh Frozen Plasma (FFP), and 30 ml Packed Red Cells (PRC) transfusion. On the way, aged 19 h, the baby experienced many hematemeses. At 26 h, meconium was only slightly mixed with blood. At the age of 30 h, the baby can drink well. The patient went home after PT, aPTT, and INR had decreased to the normal limit. The baby’s mother was happy that her child was safe and healthy.

## Discussion and conclusion

Early-onset VKDB in newborns is usually associated with medications taken by the mother during pregnancy. These drugs include anti-epileptic drugs (carbamazepine, phenytoin, and barbiturates), anti-tuberculosis drugs (isoniazid and rifampicin), and certain antibiotics (cephalosporins), vitamin K antagonist drugs (warfarin). Also, early-onset VKDB is associated with impaired fat and fat-soluble vitamin absorption in the mother and is often found in pregnant women who do not receive vitamin K prophylaxis before delivery [5, 11]. In Japan, a national survey from 2007 to 2016 found 18 cases of early-onset VKDB. Of the 18 mothers whose babies had early-onset VKDB, 12 were malnourished, 3 had Crohn’s disease, and three were on warfarin therapy [5]. In this case, the mother regularly had antenatal care with an obstetrician, and it was said that her pregnancy was normal. The mother did not take any medicine except vitamins that do not contain vitamin K. The mother also has never experienced fat malabsorption disorders. Prior to delivery, the mother did not experience bleeding. However, when screened, it turns out that the haemoglobin is 7 g/dL. The patient received a PRC transfusion before delivery. The delivery process was smooth, and there were no problems. So, in this case, no factors in the mother cause early-onset VKDB.

Immediately after birth, the baby received an injection of vitamin K1 1 mg IM. Then, as soon as the baby is born, the baby can breastfeed directly to her mother. Abruptly, 50 min after delivery, there was meconium with lots of fresh blood (Haematochezia). The baby still looks well done. Laboratory results showed average platelet values and normal blood morphology on a peripheral blood smear. When the baby was 7 h old, the baby had much haematochezia. Laboratory results showed decreased haemoglobin two g/dL in 6 h and increased PT, aPTT, and INR. At the time, the working diagnosis was gastrointestinal bleeding due to idiopathic early onset of VKDB. Then, the baby received vitamin K1 2 mg IM, 2 × 60 ml FFP, and 30 ml PRC transfusion.

The early-onset VKDB diagnosis, in this case, was established based on the presence of gastrointestinal bleeding in a clinically healthy newborn with no thrombocytopenia. In addition, normal blood morphology at peripheral blood smear, increased PT, aPTT, and INR, and the values of PT, aPTT and INR returned to normal after administration of vitamin K1 [5, 12–16]. Moreover, no abnormalities were found on the babygram and abdominal ultrasound.

Why did a baby receiving vitamin K1 1 mg still have early-onset VKDB? Factors inducing vitamin K deficiency in newborns are a poor placental transfer of vitamin K, immature gut flora, low vitamin K content in breast milk and substantial differences among individuals, poor intestinal absorption of vitamin K, and the low activity level of vitamin K epoxide reductase [11, 17]. In this case, the cause may be a poor placental transfer of vitamin K. The others cause low vitamin K content in breast milk or poor intestinal absorption of vitamin K. In healthy newborns, vitamin K1 in cord blood is often below the detection limit of 0.02 ug/L [18]. The content of vitamin K1 in mature human milk is only 2.1 ug/L, and in colostrum, 2.3 ug/L. While formula milk can be up to 4.9 ug/L [19]. Even if taking mature human milk at 200 mL/kg a day, it has been estimated that a baby would receive less than 1 µg vitamin K a day [16]. One study showed that 25 ug of vitamin K3 is the minimum effective dose required to achieve optimal prothrombin complex levels in term newborns [20]. An injection of 1 mg of vitamin K1 at birth will cause vitamin K1 levels 24–48 h later to reach 1000–5000 times higher than vitamin K1 levels at birth [16]. Other researchers reported that vitamin K1 1–2 mg IV or SC would normalize the coagulation profile within two to three hours [4, 11, 16]. This can explain why at 50 min, the baby has early-onset VKDB because the prothrombin complex level has not yet reached the optimal level to prevent bleeding.

Vitamin K1 1–2 mg (250–300 µg/kg body weight) IM or SC should be given immediately to treat early-onset VKDB. For severe bleeding episodes, FFP or prothrombin complex concentration may be administered in addition to vitamin K [14, 21]. FFP in a dose of 15 mL/kg or prothrombin complex concentrates 50 units/kg body weight. FFP administration may be repeated every 8 to 12 h, depending on the infant’s needs. rFVIIa can also be effective in life-threatening hemorrhage at 90 g/kg [16]. In this case, vitamin K1 2 mg IM, 2 × 60 mL FFP, and 30 ml PRC transfusion were given because of heavy bleeding. After being given the above therapy, 23 h later, the baby returned home in stable condition.

Early-onset VKDB, of course, can be prevented. Pregnant women taking drugs that can interfere with vitamin K metabolism, have fat malabsorption, or are about to give birth preterm should be given prophylactic 15–30 mg of vitamin K daily 2 to 4 weeks before delivery. After giving an oral dose of 20 mg of vitamin K1, the concentration of vitamin K1 in the breast milk of one mother rose to 140 ug/L after 12 h. At 48 h, it was still about twice the average endogenous level of human milk. One study showed that babies born to mothers who received vitamin K 20 mg orally, a single dose, 4–24 h before expected delivery, had higher prothrombin levels than babies whose mothers did not receive vitamin K. Then, immediately after birth, the baby is given 1 mg of vitamin K1 IM. Several studies suggest that infants at high risk, such as preterm infants, and babies with liver disorders, should be given a vitamin K1 booster [19, 21, 22]. A systematic review study and meta-analysis concluded that antenatal administration of vitamin K1 to a mother would increase vitamin K1 levels in maternal plasma, newborn plasma, breast milk, and maternal-newborn PIVKA-II factor. Therefore, especially vitamin K1 supplementation should be given to mothers that intake anti-epileptic drugs, anti-tuberculosis drugs, and vitamin K antagonist drugs [23]. The limitation of this case report is that the levels of clotting factors II, VII, IX, and X cannot be checked.

Vitamin K1 1 mg IM prophylaxis should be given immediately after birth to prevent early-onset VKDB. In addition, pregnant women who received drugs that interfere with vitamin K metabolism (anti-epileptic drugs, anti-tuberculosis drugs, vitamin K antagonist drugs) or have fat malabsorption should be given prophylactic vitamin K1, 20 mg/day orally. Vitamin K1 should be given at least two weeks before the expected delivery time.

## Data Availability

The datasets used and analyzed during the current study are available from the corresponding author upon reasonable request.

## Abbreviations

VKDB: Vitamin K deficiency bleeding
FFP: Fresh Frozen Plasma
PRC: Packed Red Cells
PT: Prothrombin Time
aPTT: Activated partial thromboplastin time
INR: International Normalized Ratio
PIVKA-II: Protein induced by vitamin K absence or antagonist II
